# A Respiratory Motion Estimation Method Based on Inertial Measurement Units for Gated Positron Emission Tomography

**DOI:** 10.3390/s21123983

**Published:** 2021-06-09

**Authors:** Eero Lehtonen, Jarmo Teuho, Juho Koskinen, Mojtaba Jafari Tadi, Riku Klén, Reetta Siekkinen, Joaquin Rives Gambin, Tuija Vasankari, Antti Saraste

**Affiliations:** 1 Department of Computing, University of Turku, 20014 Turku, Finland; juanko@utu.fi (J.K.); reetta.siekkinen@tyks.fi (R.S.); joaquin.j.rives@utu.fi (J.R.G.); 2 Turku PET Centre, University of Turku and Turku University Hospital, 20521 Turku, Finland; jarmo.teuho@tyks.fi (J.T.); riku.klen@utu.fi (R.K.); antsaras@utu.fi (A.S.); 3 School of ICT, Faculty of Engineering, Turku University of Applied Sciences, ICT-City, Joukahaisenkatu 3, 20520 Turku, Finland; mojtaba.jafaritadi@turkuamk.fi; 4 Department of Medical Physics, Turku University Hospital, 20521 Turku, Finland; 5 Heart Centre, Turku University Hospital, 20521 Turku, Finland; tuija.vasankari@tyks.fi

**Keywords:** microelectromechanical systems, motion estimation, positron emission tomography, signal processing

## Abstract

We present a novel method for estimating respiratory motion using inertial measurement units (IMUs) based on microelectromechanical systems (MEMS) technology. As an application of the method we consider the amplitude gating of positron emission tomography (PET) imaging, and compare the method against a clinically used respiration motion estimation technique. The presented method can be used to detect respiratory cycles and estimate their lengths with state-of-the-art accuracy when compared to other IMU-based methods, and is the first based on commercial MEMS devices, which can estimate quantitatively both the magnitude and the phase of respiratory motion from the abdomen and chest regions. For the considered test group consisting of eight subjects with acute myocardial infarction, our method achieved the absolute breathing rate error per minute of 0.44 ± 0.23 1/min, and the absolute amplitude error of 0.24 ± 0.09 cm, when compared to the clinically used respiratory motion estimation technique. The presented method could be used to simplify the logistics related to respiratory motion estimation in PET imaging studies, and also to enable multi-position motion measurements for advanced organ motion estimation.

## 1. Introduction

Patient motion is a major source of image blurring in positron emission tomography (PET) studies [1,2,3,4]. Image quality in, for example, cardiac PET, is reduced by respiratory and cardiac motions due to the relatively long acquisition time of PET images. Spatial resolution of PET can be improved by estimating and compensating for the motion of the heart and the respiratory motion in a process called gating [1,2,3,4]. In gating the acquired PET data are divided into disjoint sets, known as bins, depending on the phase or amplitude of the estimated motion. In respiratory gating, the PET data are divided according to a respiratory signal, whereas in cardiac gating, a heart-related signal (for example, electrocardiography (ECG) or seismocardiography) is used. In dual-gating both a respiratory and a cardiac signal are used together to divide the PET data into bins.

In a clinical setting, the respiration signal for gated PET is typically obtained by using piezo-electric respiratory belts, infrared optical systems, spirometers, nasal temperature and humidity sensors, or by using data-driven methods which estimate respiration directly from the PET data [1,4]. Each of these techniques have their advantages and drawbacks. Using a sensor which measures directly the airflow may be uncomfortable for cardiac and respiratory patients, and optical marker-based solutions may be time-consuming to set up [1], and may result in rejected data acquisition periods due to loss of tracking. On the other hand, data-driven approaches require sufficiently high uptake of a PET radiotracer to function robustly.

In this work, we present a new body motion measurement system which uses inertial measurement units (IMUs) based on microelectromechanical systems (MEMS) technology. The measurement system contains four sensor nodes, which can be used to estimate intrafraction motions simultaneously from different locations on the body. We fuse motion signals from the sensor nodes to obtain a representation of the respiratory motion of the chest. This motion estimation is then used to gate PET data in order to mitigate respiratory motion effects in the reconstructed images. Our aims were to investigate the benefits of multinodal measurements in estimating respiratory motion, and to present a method which could simplify the logistics of sensor-based respiratory motion estimation in gated PET imaging.

### Previous Works

The amount of literature related to MEMS and IMU-based respiratory motion estimation for gated PET studies is limited, and therefore in the following we consider the most notable publications on MEMS-based general-purpose breathing frequency and respiratory motion estimation methods. Respiratory rate and tidal volume are highly informative and important vital signs [5], and there are several—both contact-based and contactless—methods to measure them. However, these methods have not been found to be sufficiently easy to use and reliable for routine use in the clinical setting [5]. Therefore, there is need for further development of respiratory motion measurement technology, and in particular, the use of MEMS motion sensors has been investigated intensively in the recent years; for a selection of recent and notable papers on this subject, cf. [6,7,8,9,10,11,12,13,14,15,16,17,18,19,20].

Using MEMS motion sensors for respiration monitoring is appealing, as the clinically used solutions are typically invasive and obtrusive, and are not suitable for prolonged monitoring outside the clinical setting [6], whereas MEMS motion sensors are non-invasive and can be used, for example, during exercise. Moreover, MEMS sensors are sensitive, accurate and low-cost, and enable simultaneous real-time measurement of motion from multiple locations on the body [5]. The main disadvantage of MEMS sensors in respiration monitoring is their vulnerability to motion artifacts [5].

The literature on MEMS-based respiration monitoring can be divided into three subsets based on which sensors are considered. Some ([7,8,9,10]) articles consider only the use of a single accelerometer to estimate the breathing rate or the respiratory motion. More recent articles typically consider using an IMU which contains an accelerometer and a gyroscope ([11,12,13,14,15,16]), or an accelerometer, a gyroscope and a magnetometer ([6,17,18]). In addition to these, a method for estimating respiratory motion using only a magnet and a MEMS magnetic sensor is presented in [19]. The number of MEMS sensors used form estimating respiratory motion varies between studies. Methods utilizing multiple IMU sensors apply common mode filtering [6,13,14,17,20], independent component analysis (ICA) [7], or a kinematic joint model [18] to achieve position independence or to mitigate motion artifacts during exercise.

Various sensor fusion methods are used to combine information from different types of sensors and different sensor nodes. Common sensor fusion methods include the principal component analysis (PCA) [17,21], ICA [7,21], Kalman filters [12,19], and a pose estimation method [22] developed specially to fuse signals obtained from IMU and MARG sensors [6,17]. Nearly all of these studies concentrate on estimating the 3D orientations of the sensor nodes. For example, if an IMU is attached to the chest of a subject, it is used to measure the changes of the thoracic angle according to the phase of the respiration cycle. Such a measurement is sufficient for estimating the breathing rate (including inspiratory and expiratory time) [6,13,17], flow waveform [7], and relative changes in tidal volume [14], but it cannot be used to measure the absolute position of the sensor in relation to a fixed zero level without additional assumptions (such as rigid joints in the sensor constellation [18]). In fact, to the best of the authors’ knowledge, in the current literature there is only one article [11] besides this work, which has considered the estimation of absolute respiratory motion using a MEMS motion sensor. In [11], the capability of a MEMS-based IMU to estimate the period and amplitude of the sinusoidal motion of a one-dimensional motion phantom was investigated. Two distinct methods were presented, one of which was based on double-integration of the accelerometer’s signal to obtain a position signal, and the other on integrating the gyroscope’s angular velocity signal, and then multiplying the obtained angular signal by the estimated radius of motion. The accelerometer method was found to be more accurate at estimating the period of the motion, whereas the gyroscope method was more accurate at estimating the magnitude of the motion. It should be noted that a correct radius of motion must be defined in order for the gyroscope method to work properly.

In our research we have investigated the usefulness of MEMS-based motion estimation to the gating of PET imaging. The idea of MEMS gating was first presented in [9], which considered the use of a MEMS accelerometer for generating signals for PET dual gating. In [23] this method was extended to use both MEMS accelerometers and gyroscopes, and as a clinical application, MEMS gating of cardiac PET for two atherosclerotic patients was demonstrated. We improved the methodology’s robustness and accuracy further by applying PCA to respiration filtering and ICA to cardiac filtering in [21]. The usefulness of MEMS sensors in the gating of medical imaging is based on the sensitivity of the sensors, on their high sampling rate, and on the possibility to simultaneously measure motion from several locations on the body. A motion estimation patch based on MEMS technology could mitigate some of the typical logistic problems of optical marker-based solutions.

The presented work is a continuation of our previous research [9,21,23]. In this work we present a new multi-sensor measurement system for estimating the sensors’ positions, and thus, the polarity and magnitude of the respiratory motion, in order to perform amplitude-based gating in PET imaging. Measurement signals acquired from two sensor nodes are considered for the respiratory motion estimation. The signals obtained from one of these nodes is processed using PCA, while the signal obtained from the other node is processed using Madgwick’s pose estimation method [22]. The resulting signals are fused in order to obtain a respiratory motion signal, which is compared against a ground truth signal obtained using an optical marker-based solution (real-time position management (RPM) by Varian Medical Systems, Inc., Palo Alto, CA, USA). In comparison, in our previous works [9,21,23], we considered only a single MEMS-based IMU for respiratory motion estimation. It should also be noted that the presented method is the first reported IMU-based method which can measure the magnitude and phase of the respiratory motion without any extra assumptions of the radius of motion. As a novel application of IMU-based motion estimation, the acquired respiratory motion signals were used for gating in [${}^{68}$Ga]NODAGA-RGD PET studies [24], and the resulting PET images were compared to RPM respiratory-gated images in terms of several different image quality metrics. Although the motivation for this work was to further develop motion estimation methodology for gated PET studies, the presented method itself is general, and could be used in other applications where respiratory rate and respiratory motion magnitude are of interest.

## 2. Materials and Methods

### 2.1. Hardware: A Multinodal Motion Measurement System

**Figure 1:**
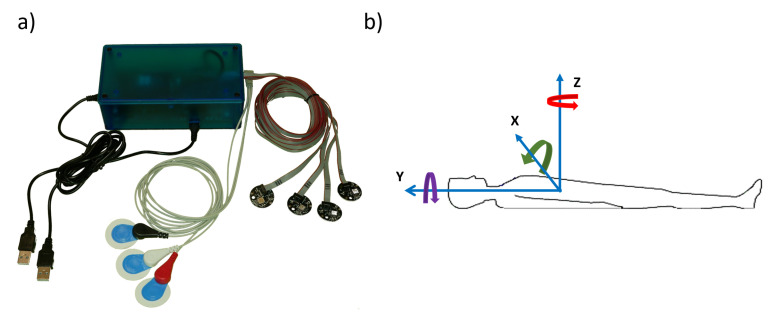
The motion measurement system (MMS) and the directions of measurement axes. Inset (**a**): the novel multinodal measurement system, which consists of four MEMS-based motion measurement nodes and three ECG electrodes. Inset (**b**): directions of the measurement axes with respect to the measured subject.

Our new multinodal *Motion Measurement System (MMS)*—depicted in Figure 1a—consists of a central unit, and four peripheral MEMS sensor nodes and three ECG electrodes. Each of the MEMS sensor nodes contains a BMI160 IMU unit (Bosch Sensortec GmbH, Reutlingen, Germany) and a three-axis ADXL355 accelerometer (Analog Devices, Inc., Wilmington, MA, USA). Each BMI160 unit contains a 3D accelerometer and a 3D gyroscope. It should be noted that in this work we only consider sensors and not actuators based on MEMS technology—that is, we only intend to measure motions and changes in positions and orientations, and not to induce any such changes. In the following we used the data acquired from the accelerometers of the ADXL355 sensors and the gyroscopes of the BMI160 sensors, since the noise floor of the ADXL355 sensor is significantly lower than that of the BMI160 accelerometer. The data from the BMI160 sensor were acquired at the sampling rate of 400 Hz; the acquisition range for the gyroscope was ±125 degrees per second. The data from the ADXL355 sensor were acquired with the sampling rate of 500 Hz, with the acquisition range of ±2 g (where g is the gravitational acceleration). The central unit of the measurement system is based on the Nordic Semiconductor nRF52840 development kit. The central unit takes care of the communication to and from the sensor nodes and electrodes, and encapsulates the data samples into packets. The measurement system sends timestamped packed binary data through one megabaud serial connection via USB connection to the PC, where the data were saved. The created file containing binary packets was then converted to SI units with timing information, and stored in a Matlab MAT-file for further processing using Matlab or Python.

The PC was synchronized with the PET system at the beginning and end of each gated PET study. However, the clock signal that can be obtained from the PET scanner for the synchronization procedure had only a one-second resolution. Therefore, in this work we fine tuned the synchronization by manually finding a suitable timing offset which makes the raw MMS signal (the *x* or *y*-channel of the sensor node 1) coincide with the RPM signal. In future we may avoid this manual procedure either by establishing a method to acquire a higher resolution clock signal from the PET scanner, or by performing signal synchronization by matching the hospital ECG with the ECG acquired by our measurement system.

### 2.2. MMS Measurement Locations

We used two of the available four MMS sensor nodes to estimate the respiratory motion. The other two sensor nodes of our measurement system can be used, for example, to obtain cardiac motion information, and to measure the overall motion of the patient bed during imaging. The two respiratory motion measurement nodes—denoted as sensor nodes 1 and 2 in the following—were attached on the surface of the body by two-sided skin-friendly adhesive. Both nodes were attached to the skin at the center line of the ventral side of the body—node 1 on the xiphoid process of the sternum, and node 2 on the abdomen, superior to the umbilicus. The orientation of each MEMS motion sensor with respect to the measured subject’s anatomy was as follows: The *x*-axis pointed dexter to sinister, the *y*-axis caudal to cranial and the *z*-axis dorsal to ventral, as depicted in Figure 1b.

The choices of measurement locations can be justified as follows. Firstly, in previous studies [7,17] it was found that the best thoracic inclination signal is obtained near the position of node 1. Secondly, reliable respiratory signals were previously acquired from the abdomen near the position of node 2 in [17], and it was also close to where the RPM marker block is typically located.

### 2.3. Positron Emission Tomography Study

#### 2.3.1. Subject Details

Eight subjects who underwent a combined [${}^{15}$O]$H_{2}O$ and [${}^{68}$Ga]NODAGA-RGD (RGD) positron emission tomography/computed tomography (PET/CT) protocol [24] were recruited to the study. Population characteristics are summarized in Table 1. The subjects underwent imaging 3 to 14 days after an ST-elevation myocardial infarction. Each subject gave an informed consent. The study was approved by the ethics committee of the Hospital District of Southwest Finland and Turku University Hospital, and the study was conducted in accordance with the Declaration of Helsinki.

#### 2.3.2. PET/CT System

All PET data were acquired on the Discovery MI PET/CT system with a 4-ring configuration. The Discovery MI has 4 rings of silicon photomultiplier (SiPM) detectors. One detector block comprises 4 × 9 array of 3.95 × 5.3 × 25 mm LBS-crystals coupled to a 3 × 6 array of SiPM with 2 × 3 pixels. Transaxial and axial fields of view (FOV) were 70 cm and 20 cm, respectively. The coincidence timing and energy windows were 4.9 ns and 425–650 keV, respectively. The system’s performance details are described in detail in [25].

#### 2.3.3. PET/CT Imaging Protocol for RGD

For PET attenuation correction and anatomical localization, a low-dose helical CT was acquired with automatic dose modulation. The helical rotation time was 0.5 s with 3.75 mm thickness. A tube voltage of 120 kV and tube current with range of 15 to 180 mA were used. The tube current was modulated automatically according to the noise index, which was 28.50. The CT was collected in non-gated mode and the patient was instructed to breathe freely during the CT acquisition.

PET data were acquired in list-mode for one bed position over the heart, after an uptake period of 60 min of the [${}^{68}$Ga]NODAGA-RGD. 3D-PET with simultaneous respiratory and cardiac gating for 15 min was acquired. Respiratory position monitor (RPM) system (Varian Medical Systems, Palo Alto, CA, USA) was used for clinical respiratory motion tracking. A standard 3-lead ECG system (IVY-3150, Ivy Biomedical Systems, Inc., Branford, CT, USA) integrated with the PET/CT hardware was used for cardiac gating. MMS signals were acquired during from the measurement locations described in Section 2.2.

#### 2.3.4. PET/CT Imaging Protocol and Image Reconstruction for [${}^{15}$O]$H_{2}O$

Similarly to RGD, a low-dose helical CT was collected using a tube voltage of 120 kV; a tube current range of 10 to 120 mA; a noise index of 30.00; and the rotation time and thickness of 0.5 s and 3.75 mm, respectively. The CT was collected in non-gated mode and the patient was instructed to breathe freely during the CT acquisition.

PET was collected in list-mode with acquisition duration of 4 min and 40 s. The image acquisition was started from 35 s from the injection of O–15 water. The target dose was 500 MBq for all subjects. Images were reconstructed directly on the PET/CT system using the time-of-flight block sequential regularized expectation maximization (TOF-BSREM, vendor name: Q.Clear) with a beta value of 350. Dynamic frame times of 14 × 5 s, 3 × 10 s, 3 × 20 s and 4 × 30 s were used. The matrix size and FOV were 192 × 192 × 71 and 350 mm. All quantitative corrections, including the all calibrations, randoms, scatter and attenuation correction, were applied to the PET data.

#### 2.3.5. Gated PET Image Reconstruction for RGD

PET list-mode data, respiratory and cardiac triggers and CT images for attenuation correction were exported from the Discovery MI PET/CT system. In addition, the respiratory signals from the MMS and the RPM were extracted. For PET reconstruction, a vendor-provided reconstruction toolbox was used (Duetto, version 2.01) with in-house developed Matlab scripts for gating. All image reconstructions were performed with Matlab version R2017a (The Mathworks, Inc., Natick, MA, USA). In short, the gating and reconstruction pipeline involves respiratory signal time synchronization to list-mode data, definition of gate timings based on the respiratory signal and unlisting of gated sinograms followed by image reconstruction. All gated reconstructions for RPM and MMS were performed using respiratory gating. For comparison, a non-gated reconstruction using the entire acquisition time of 15 min was performed. For respiratory gating, amplitude gating with 5 equidistant gates were used. The maximum and minimum gating thresholds were defined globally defined on the upper and lower 20% quantiles (0.2 to 0.8) of the MMS and RPM signals. For gating, only the cycles considered valid by the RPM system were used for both the MMS and the RPM signals to ensure that the same cycles are rejected for both signals for fair comparison. PET images were reconstructed using the TOF-BSREM algorithm, with a beta value of 500. The matrix size and FOV were 192 × 192 × 71 and 350 mm. All quantitative corrections including all calibrations, randoms, scatter and attenuation correction were applied to the PET data.

#### 2.3.6. PET Data Preservation

The amount of data preserved compared to the non-gated image (100%) was calculated for all methods. For both MMS and RPM, we assessed the amount of data lost due to cycle rejection or signal inconsistencies and the amount of data preserved in each respiratory gate. We report the data saved in each gate for MMS and RPM with the amount of data rejected as mean ± standard deviation across all patients.

#### 2.3.7. PET Image Analysis

PET image analysis was done with Carimas 2.9 software (Turku PET Centre, Turku, Finland) using the analysis protocol described in [24]. After visualization and definition of the heart long axis, the myocardial volumes of interest (VOIs) in the [${}^{15}$O]$H_{2}O$ images were semi-automatically defined. The VOIs contained the whole left ventricle and a blood pool as separate volumes. These contours were then imported to overlapping [${}^{68}$Ga]NODAGA-RGD PET images from non-gated, RPM-gated and MMS-gated reconstructions followed by visual confirmation of the overlap of contours and appropriate anatomical detail. For the RPM and the MMS, the gated images from bin number 5 (end-expiratory bin) were used in the analysis as they contained the best image quality. After visual confirmation, the contour locations needed further manual adjustment for subjects 4 and 6 to match the contours for the myocardial region in non-gated, MMS-gated and RPM-gated reconstructed PET. From both the myocardium and blood pool, the mean and standard deviation in PET uptake (Bq/mL) was then defined. For assessment of image quality, contrast ratio (CR) [26,27], signal-to-noise ratio (SNR) [28], coefficient of variation (CV) [28] and contrast-to-noise ratio (CNR) [26,27] were used. The uptake values were used to calculate the image quality metrics from each PET image series as follows:

(1) $$\mathrm{CR}=\frac{{\mathrm{Myocardium}\mathrm{VOI}}_{\mathrm{mean}}}{{\mathrm{Blood}\mathrm{pool}\mathrm{VOI}}_{\mathrm{mean}}}$$

(2) $$\mathrm{SNR}=\frac{{\mathrm{Myocardium}\mathrm{VOI}}_{\mathrm{mean}}}{{\mathrm{Blood}\mathrm{pool}\mathrm{VOI}}_{\mathrm{sd}}}$$

(3) $$\mathrm{CV}=\frac{{\mathrm{Myocardium}\mathrm{VOI}}_{\mathrm{sd}}}{{\mathrm{Myocardium}\mathrm{VOI}}_{\mathrm{mean}}}$$

(4) $$\mathrm{CNR}=\frac{{\mathrm{Myocardium}\mathrm{VOI}}_{\mathrm{mean}}-{\mathrm{Blood}\mathrm{pool}\mathrm{VOI}}_{\mathrm{mean}}}{{\mathrm{Blood}\mathrm{pool}\mathrm{VOI}}_{\mathrm{sd}}},$$

where ${\mathrm{VOI}}_{\mathrm{sd}}$ is the standard deviation and ${\mathrm{VOI}}_{\mathrm{mean}}$ is the average uptake in the corresponding volume of interest.

In addition to the image quality metrics, to evaluate the image quality of the PET images with RPM and MMS gating, manually selected profiles over a hot spot for gated images were extracted.

### 2.4. Respiratory Motion Estimation Algorithm for the MMS

**Figure 2:**
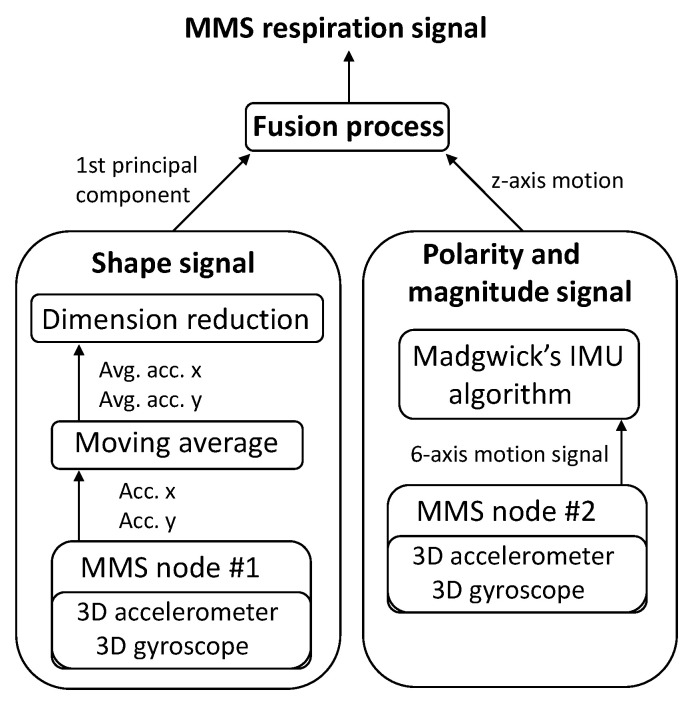
Flow diagram of the respiration motion estimation algorithm.

The respiratory motion estimation algorithm presented in this work is based on fusing two motion signals together. On one hand, we apply PCA to obtain the shape of the respiration signal based on the signal measured by the sensor node 1, and on the other hand, we apply a linear *z*-axis motion estimator on the signal acquired by the sensor node 2 to obtain the polarity and the magnitude of the respiration signal. Figure 2 illustrates the high-level flow of the proposed respiration motion estimation algorithm.

The data acquired from the sensors is downsampled to the sampling rate of $f_{s}=$ 100 Hz, so that for each timestamp we have a unique sample from each of the nodes and axes of the BMI160 gyroscope and the ADXL355 accelerometer. In the following we describe the working principles of the shape signal block and the polarity and magnitude signal block, and how they are fused together to form a respiratory motion signal.

#### 2.4.1. Shape Signal

The respiration motion shape is obtained from the signal measured by node 1. It has been previously noted [21] that the thoracic angle signal has similar shape as the RPM signal. To obtain a thoracic angle signal, we process the *x*- and *y*-axis channels of the ADXL355 accelerometer of node 1.

**Figure 3:**
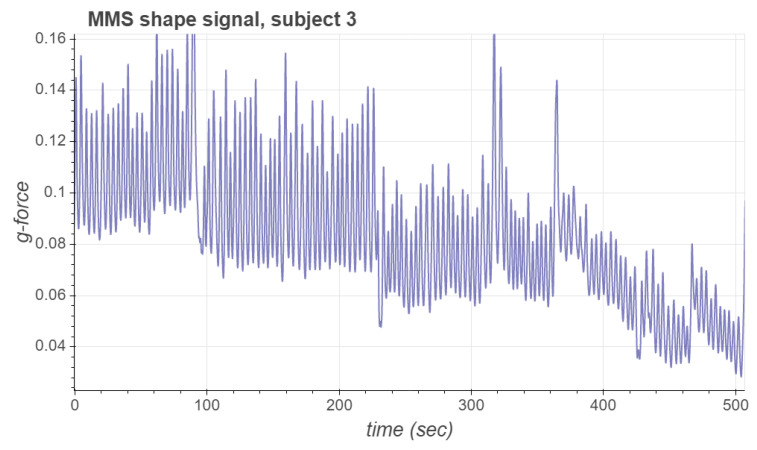
MMS shape signal for subject 3.

The input signals are first filtered by a moving average filter, whose window length is $w_{\mathrm{avg}}^{S}$. The resulting averaged signals are stored into a circular buffer of length $N_{\mathrm{circ}}$. Once the circular buffer is full, a PCA basis is computed from the two-dimensional stored data. This basis is subsequently used to obtain the first principal component of the averaged signals, which we call the *shape signal* for the respiration motion estimation. As an example, Figure 3 presents the shape signal for subject 3. In Table 2 we list the values of the parameters used for the numerical results of this work. Notice that the polarity of the first principal component can be positive or negative with respect to the RPM signal. In [21] we used the correlation between the *z*-axis acceleration signal and the first principal component to deduce the correct polarization; however this method was not reliable with the data considered in this work. A more robust way of computing the polarity is explained in the following subsection.

#### 2.4.2. Polarity and Magnitude Signal

In agreement with [7], we have found that largest respiratory motion can be registered in the abdomen area near diaphragm. Therefore, the *polarity and amplitude* signals are obtained by processing the signals acquired by node 2. For these signals, we used the accelerometer signals acquired by the ADXL355 sensor and the gyroscope signals acquired by the BMI160 sensor.

**Figure 4:**
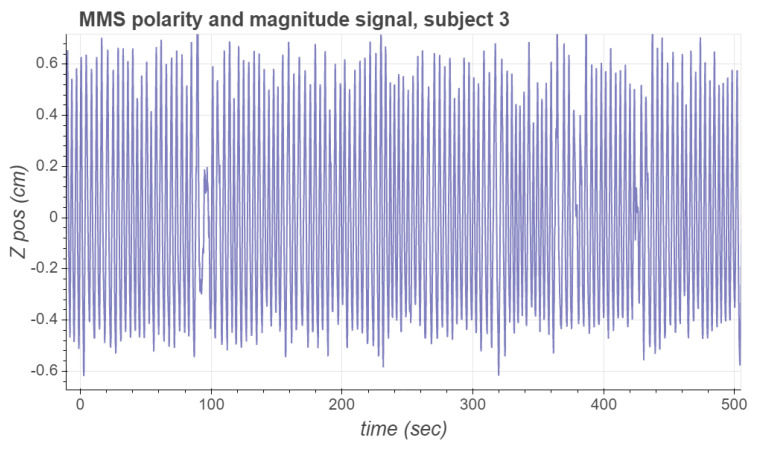
MMS polarity and magnitude signal for subject 3.

This six-dimensional input signal is given as an input to Madgwick’s IMU filter, which is described in detail in [22]. The acceleration $a_{z}$ in the direction of the *z*-axis in the world coordinates (upwards as the subject is assumed to be in supine position during the measurement) is estimated by the IMU filter, which yields the respiratory acceleration as

(5) $$a_{\mathrm{resp}}=a_{z}-g,$$

where *g* is the value of the gravitational acceleration. The respiratory acceleration is then numerically integrated as a cumulative sum weighted by the time between successive samples, and high-pass filtered using a second-order Butterworth filter with a cutoff frequency $f_{B}$ to obtain an estimate of the respiration velocity. The velocity signal is again numerically integrated and high-pass filtered with a second-order Butterworth filter with the same cutoff frequency $f_{B}$ to obtain an estimate of the respiration position. Figure 4 presents the polarity and magnitude signal for subject 3.

**Figure 5:**
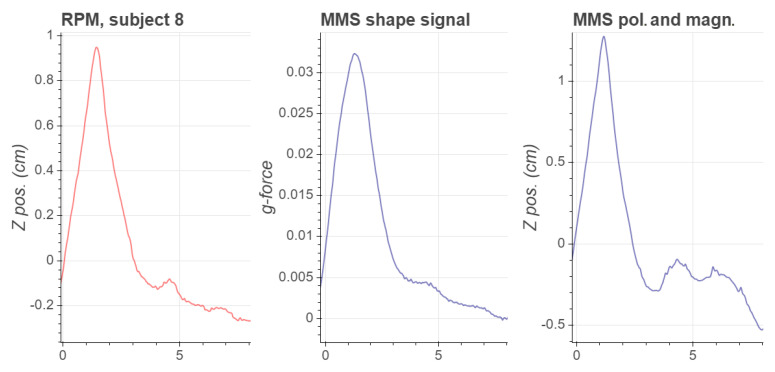
RPM and MMS waveforms corresponding to a single respiratory cycle at scan time *t* = 0 s–8 s for subject 8.

The high-pass filters are applied in order to mitigate baseline drift due to noise in the MMS signals. For periodic signals as the respiration, the described method produces an estimate of the linear position along the *z*-axis, but it should be noted that if the motion for some reason stops or becomes very slow, the high-pass filtering will force the signal towards zero. In our experiments this was not an issue, and the resulting magnitude signal corresponds relatively well to the magnitude of the RPM signal. However, the shape of this position signal is not as similar to the RPM signal as the shape signal, which is why the shape signal is computed separately. As an example, in Figure 5 we present the curves corresponding to a respiration cycle for subject 8 for the RPM, the MMS shape signal, the MMS polarity and fhe magnitude signal. It can be seen that the expiration phase of the respiratory cycle is estimated more accurately by the MMS shape signal than the polarity and magnitude signal.

#### 2.4.3. Fusion Process

The fusion of the shape signal s(t) and the polarity and magnitude signal p(t)—where *t* is assumed to take discrete values with a time step of $\Delta t=1/f_{s}$—is performed as follows. Firstly, the shape signal and the polarity and magnitude signal must be synchronized. Butterworth high-pass filtering of the magnitude signal results in an unknown and frequency dependent delay with respect to the shape signal. Furthermore, this delay can be either positive or negative, since we use a forward-backward filtering algorithm in order to mitigate the frequency dependent distortion. To resynchronize the signals, we compute their correlation over the mid-half of the signal, and determine the delay which maximizes it. More precisely, let the duration of the shape signal be *T*. The sought delay *d* is determined as

(6) $$d={\mathrm{argmax}}_{d^{\prime }}\left|\sum_{t=T/4}^{3T/4}s\left(t\right)p\left(t+d^{\prime }\right)\right|,$$

and it is found by letting *d* go through all the possible discrete values ($d=k/f_{s}$, where *k* is an integer and $f_{s}$ is the sampling rate) in the interval $\left[-d_{\max},d_{\max}\right]$. In the experimental results presented in this paper we have used $d_{\max}=2\;s$, which was sufficient for finding correct delay for the polarity and magnitude signal for all subjects. The polarity of the respiration signal *q* is then defined as

$$q=\mathrm{sign}\left(\sum_{t=T/4}^{3T/4}s\left(t\right)p\left(t+d\right)\right),$$

The magnitude of the fused respiration signal is obtained by estimating the moving average and variance of the considered signals using Welford’s formulas [29]. The moving average and variance of the signals are estimated using moving average window lengths of $w_{\mathrm{avg}}^{F1}$ and $w_{\mathrm{avg}}^{F2}$, respectively. Let us denote the corresponding moving average signals as

(7) $$s_{\mathrm{avg}}\left(t\right),\;\;s_{\mathrm{var}}\left(t\right)$$

for the shape signal, and

(8) $$p_{\mathrm{avg}}\left(t\right),\;\;p_{\mathrm{var}}\left(t\right)$$

for the polarity and magnitude signal, respectively. Then the fused respiration motion signal—without baseline correction—is defined as

(9) $$r_{\mathrm{wobc}}\left(t\right)=q\sqrt{\frac{p_{\mathrm{var}}\left(t\right)}{s_{\mathrm{var}}\left(t\right)}}\left(s\left(t\right)-s_{\mathrm{avg}}\left(t\right)\right),$$

where *q* is the polarity obtained from the correlation process.

Finally, there typically is some slow-varying baseline drift in the RPM respiration signals due to small body movements and changes in muscle tension during the imaging. We use the shape signal to estimate the baseline drift, and define

(10) $$b\left(t\right)=\left(s_{{\mathrm{avg}}^{\prime }}-\bar{s(t)}\right)\cdot \sigma_{s(t)}/\sigma_{r(t)},$$

where $s_{{\mathrm{avg}}^{\prime }}$ denotes the moving average of the shape signal with window length $w_{\mathrm{avg}}^{F3}$, $\bar{s(t)}$ is the mean value of the shape signal and σ is the standard deviation of a signal. In other words, our estimation of the baseline signal corresponds to a zero-centered and scaled version of low-pass filtered shape signal. The baseline-corrected respiration signal is then obtained as

(11) $$r\left(t\right)=r_{\mathrm{wobc}}\left(t\right)+qb\left(t\right).$$

**Figure 6:**
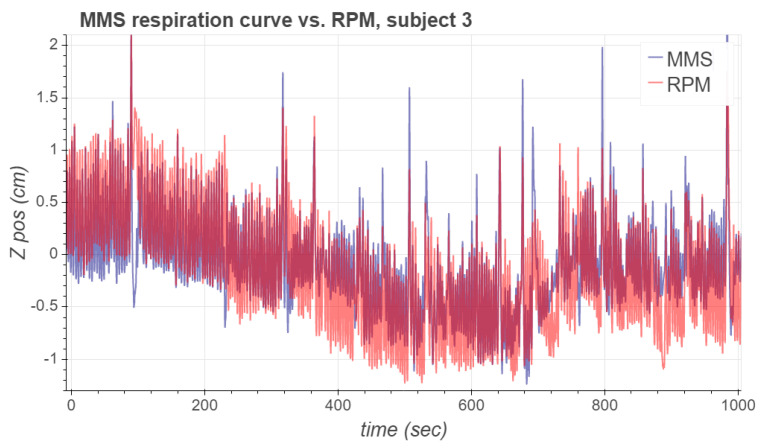
MMS and RPM respiration signals for subject 3.

Figure 6 shows a comparison between the RPM respiration signal and the baseline-corrected MMS respiration signal. Clearly, without baseline drift estimation the MMS signal could not follow the long-term trend of the RPM signal.

## 3. Results

### 3.1. Comparison Metrics

In the following, we denote the MMS respiration waveform obtained from the fusion process by $r(t_{i})$, and its first discrete difference by $r^{\prime }\left(t_{i}\right)=r\left(t_{i+1}\right)-r\left(t_{i}\right)$. The RPM waveform is denoted by $r^{\mathrm{RPM}}\left(t\right)$.

The RPM software (Varian Medical Systems, Inc., Palo Alto, CA, USA) outputs trigger timestamps, which correspond to end inspiration and end expiration times. In order to facilitate the comparison analysis, we inspected the timings of the end expiration trigger times in comparison to the RPM signal, and manually corrected erroneously placed triggers and added new end expiration triggers where they were missing. Then we used these corrected end expiration time triggers to define a search window for finding the peak inspiration times in the respiration signals. Specifically, let $t_{i}$ and $t_{i+1}$ be two successive end expiration trigger timestamps. Then the corresponding set of respiration signal local maxima is defined as

(12) $$L_{i}=\left\{r\left(t_{j}\right)|t_{i}\leq t_{j}\leq t_{i+1},\mathrm{where}r^{\prime }\left(t_{j}\right)>0\mathrm{and}r^{\prime }\left(t_{j+1}\right)\leq 0\right\}$$

Now we define the inspiration peak time as

(13) $$T_{i}=\left\{\begin{array}{cc} \underset{t_{j}}{\mathrm{argmax}}\left(L_{i}\right), & \mathrm{if}\;L\;\mathrm{is}\;\mathrm{non}-\mathrm{empty}\\ \underset{t_{j}}{\mathrm{argmax}}\left\{r\left(t_{j}\right)|t_{i}\leq t_{j}\leq t_{i+1}\right\}, & \mathrm{otherwise}. \end{array}\right.$$

In other words, the peak inspiration time is defined as the time of the largest respiration signal value, which corresponds to a local maximum (the first discrete difference of the respiration signal is zero) if such a point exists, and otherwise to be the time of the largest value of the respiration signal between $t_{i}$ and $t_{i+1}$.

This definition of peak inspiration time depends on the (manually corrected) RPM end expiration trigger timings, and its error is upper-bounded by $t_{i+1}-t_{i}$. We justify this definition by noting that the error values reported in this paper are an order of magnitude smaller than those corresponding to this upper bound. Furthermore, the topic of robust peak detection is outside the scope of this paper, and therefore the availability of RPM end expiration trigger timestamps enables a simple method to compare the MMS respiration waveform to the RPM signal.

In order to facilitate comparison between the RPM and the MMS respiratory signals, we computed timings of peak inspiration times for the RPM, and denote these by $T_{i}^{\mathrm{RPM}}$. The reason for using $T_{i}^{\mathrm{RPM}}$ instead of the RPM end inspiration trigger timestamps in the following analysis is that in this way we knew that the peak inspiration times were defined identically for both signals.

We define the *i*th *breathing cycle* for RPM as the time between inspiration peaks $T_{i}^{\mathrm{RPM}}$ and $T_{i+1}^{\mathrm{RPM}}$. Similarly, the *i*th breathing cycle for MMS is defined as the time between $T_{i}$ and $T_{i+1}$. The corresponding breathing cycle durations are defined as

(14) $$d_{i}^{\mathrm{RPM}}=T_{i+1}^{\mathrm{RPM}}-T_{i}^{\mathrm{RPM}},\;\;\;d_{i}^{\mathrm{MMS}}=T_{i+1}-T_{i},$$

while the corresponding breathing cycle magnitudes are defined as

(15) $$m_{i}^{\mathrm{RPM}}=\max\left\{r^{\mathrm{RPM}}\left(t_{j}\right)|T_{i}^{\mathrm{RPM}}\leq t_{j}\leq T_{i+1}^{\mathrm{RPM}}\right\}-\min\left\{r^{\mathrm{RPM}}\left(t_{j}\right)|T_{i}^{\mathrm{RPM}}\leq t_{j}\leq T_{i+1}^{\mathrm{RPM}}\right\}$$

and

(16) $$m_{i}^{\mathrm{MMS}}=\max\left\{r\left(t_{j}\right)|T_{i}\leq t_{j}\leq T_{i+1}\right\}-\min\left\{r\left(t_{j}\right)|T_{i}\leq t_{j}\leq T_{i+1}\right\}.$$

The mean absolute breathing rate error per minute is defined as

(17) $$\mathrm{MAE}\;f_{B}=\frac{1}{T_{\min}}\sum_{i}\frac{|d_{i}^{\mathrm{MMS}}-d_{i}^{\mathrm{RPM}}|}{d_{i}^{\mathrm{RPM}}},$$

where $T_{\min}$ is the duration of the measurement in minutes. The mean respiration cycle magnitudes for MMS and RPM respiration signals are defined as

(18) $$\mathrm{MMS}\mathrm{magn}.=\mathrm{mean}\left(m_{i}^{\mathrm{MMS}}\right),\;\;\;\mathrm{RPM}\mathrm{magn}.=\mathrm{mean}\left(m_{i}^{\mathrm{RPM}}\right),$$

respectively. Finally, the mean absolute error of amplitude of the MMS respiration signal is defined as

(19) $$\mathrm{MAE}\mathrm{of}\mathrm{amplitude}=\mathrm{mean}(|r\left(t_{j}\right)-r^{\mathrm{RPM}}\left(t_{j}\right)|).$$

### 3.2. Numerical Results

RPM and MMS signals were acquired from eight subjects undergoing [${}^{68}$Ga]NODAGA-RGD and [${}^{15}$O]$H_{2}O$ PET studies, and processed offline in Matlab (R2017a and R2020b, The MathWorks, Inc., Natick, MA, USA) and in Python 3.7.9 and Numpy 1.19.1. On the whole, 10,923 s of respiration data were acquired, comprising 2075 respiration cycles. The mean acquisition time per subject was 1365 s.

**Figure 7:**
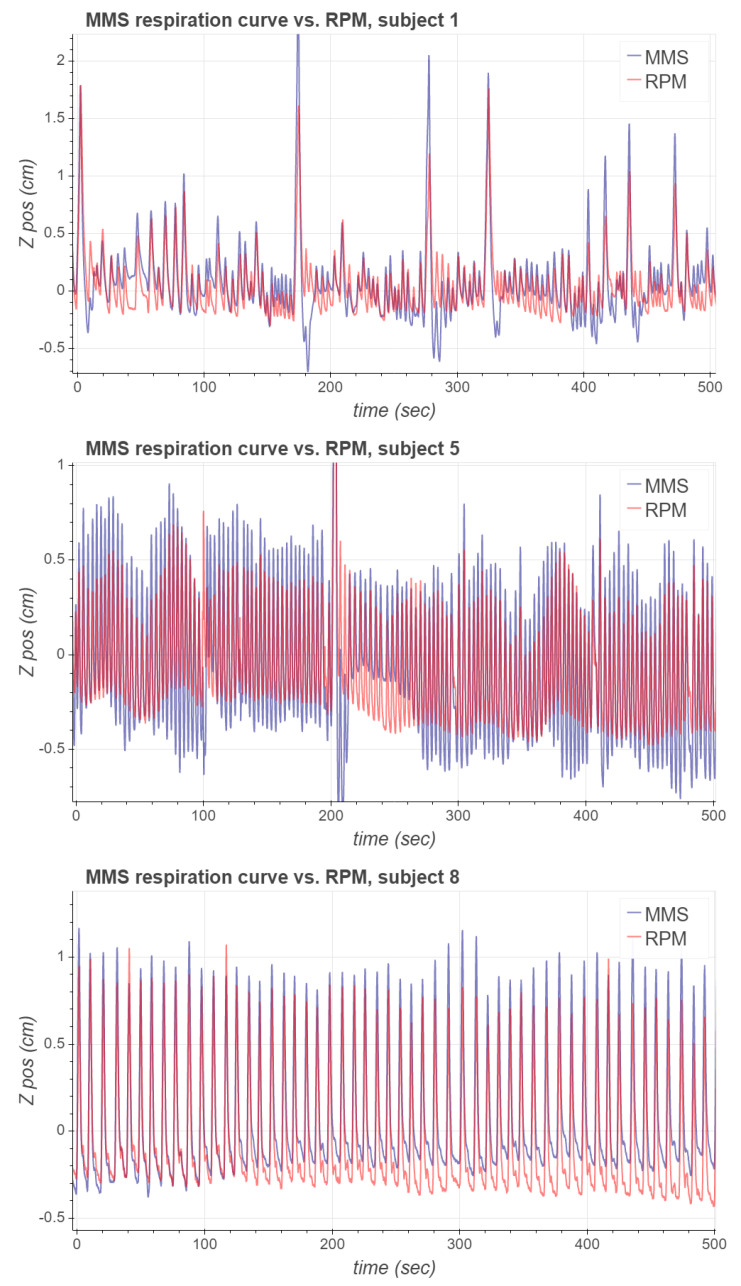
The first 500 s of the MMS and RPM respiration signals corresponding to subjects 1, 5 and 8.

To obtain the MMS respiration signal, we used the data of the first 60 s of measurement to compute the PCA basis; the first principal component was then computed for the whole acquired signal. The values for the parameters used to compute the MMS respiration signals are listed in Table 2. Figure 7 presents the acquired MMS and RPM respiration signals from subjects 1, 5 and 8 during the [${}^{68}$Ga]NODAGA-RGD PET study. These signals were chosen for visualization as they form a representative set of different types of waveforms observed in the experimental study.

As can be seen, the respiratory signal of subject 1 contains notable inspiration peaks. After some of these major peaks, it takes a few respiration cycles for the MMS signal to recover its periodicity. Respiration signals of subject 5 are more stable, and the MMS signal’s phase seems to align very well with RPM. However, the magnitude of the MMS signal is approximately 30% greater than the magnitude of the RPM signal. A possible reason for this is that the RPM marker could have been located at a position on the body which moved less due to respiration than the MMS sensor node. For subject 8, both the phase and the magnitude of the MMS respiration signal seem to match well with the RPM respiration signal, but there is a slight baseline difference between the signals.

Using the metrics defined in Section 3.1, we computed quantitative comparison results to assess the similarity of the MMS respiration signal with the RPM signal. Table 3 lists the mean signal magnitudes for the two signals, the mean absolute error of the MMS signal and the mean absolute breathing error rate per minute for the MMS signal.

The breathing error rates obtained with the presented method yielded similar results as the best methods found in the literature, as can be seen from Table 4.

### 3.3. PET Gating Results

PET gating statistics are summarized in Table 5 and Table 6. The average amounts of data preserved in both MMS and RPM were similar. Small variations were seen in standard deviation in gates 1 and 5, which represent the opposite extrema of the respiratory cycle. Individual variations can be seen across the whole group in single gates.

Image quality metrics are summarized in Table 7, Table 8 and Table 9. Image quality-wise, both MMS and RPM achieved similar CR, CV and CNR. The average SNR for MMS was slightly lower with a slightly higher CV, which is in line with the slightly lower gating statistics in bin number 5 for MMS. CR for MMS and RPM was improved in five patients, and the CNR was consistently improved for both MMS and RPM for all patients compared to non-gated images.

**Figure 8:**
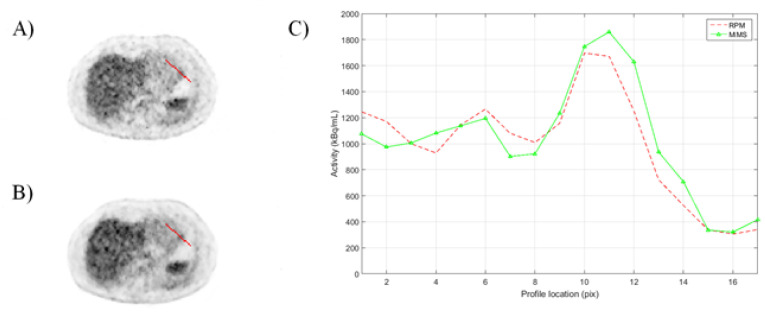
A visualization of (**A**) MMS-gated, (**B**) RPM-gated reconstructed PET images showing the region of the hot spot and (**C**) profiles between the MMS-gated and RPM-gated reconstructed images.

Finally, Figure 8 shows an example profile of subject 5, comparing the accumulation in a hot spot between RPM-gated and MMS-gated reconstructions.

## 4. Discussion

### 4.1. Logistics and the Attachment of Sensor Nodes

**Figure 9:**
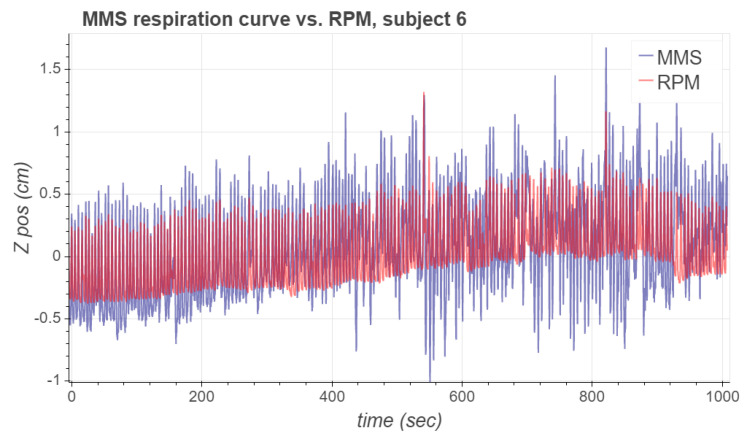
MMS vs. RPM respiratory waveforms for subject 6. The MMS signal quality deteriorates significantly in the second half of the measurement due to poor attachment of the sensor nodes.

A potential benefit of using the presented motion measurement system for PET respiratory gating is that the preparations required for signal acquisition are simpler than those required by an optical system, where one needs to verify that the camera is successfully tracking the optical marker. However, one needs to be careful when contacting the MMS sensor node to the skin in order to establish a sustained attachment; for example, it may be necessary to remove body hair under the measurement node. If the sensor nodes become even partially unattached to the skin during the measurement, the signal quality will deteriorate significantly. This was the case with subject 6, whose MMS-based respiration waveform became relatively noisy after approximately midpoint of the measurement, as is depicted in Figure 9. This was the only attachment problem we witnessed in the measurements, and despite the deterioration of the signal quality, we used the whole signal when computing the comparison metrics presented in Section 3.

### 4.2. Baseline Drift in Respiratory Signals

A challenge with the presented respiration motion estimation method is that the baseline changes in the respiration signal are difficult to estimate accurately. The main reason for this is that the polarity and amplitude signal is high-pass filtered, and therefore it does not contain information on long-term baseline changes. In this work we used the shape signal to estimate baseline drift, but there is clearly room for improvement. Furthermore, as can be seen from Table 3, the MMS signal magnitude does not perfectly match the RPM signal magnitude. If the MMS respiration signal before baseline correction is calibrated by multiplying it by the positive subject-dependent constant

γ=RPMmagn./MMSmagn.,

then the mean respiration cycle magnitudes of the MMS and RPM respiration signals will be equal.

**Figure 10:**
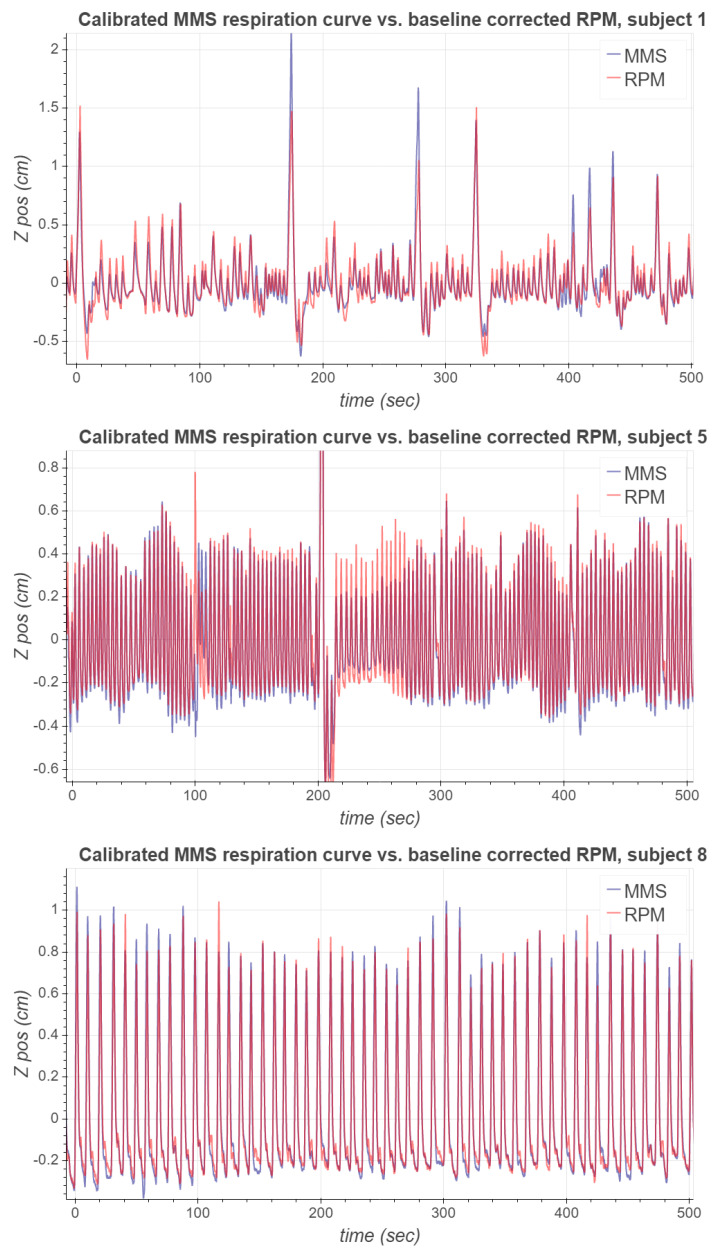
The first 500 s of the calibrated MMS and the baseline drift-corrected RPM respiration signals corresponding to subjects 1, 5 and 8.

Figure 10 presents a comparison between the calibrated MMS respiration signal without baseline estimation, and a baseline drift -corrected RPM signal for subjects 1, 5 and 8. Baseline drift correction for RPM was performed by subtracting from it its moving average with the window length of 60 s. Clearly, the respiration signals after calibration and baseline drift correction are more similar to each other than the original signals shown in Figure 7. In Table 10 the mean absolute signal errors between the original RPM and MMS signals and the baseline drift-corrected RPM and calibrated MMS signals are presented. For each subject, the absolute error became significantly lower by applying baseline drift correction and amplitude calibration.

Amplitude calibration of the MMS signal can be justified in two ways. Firstly, suppose that a subject undergoes two separate PET scans, and that in one of the scans both the RPM and the MMS respiration signals are acquired, while in the other one only the MMS signal is acquired. Then the calibrated MMS signal can be used to emulate the RPM signal for the other scan. Such is the case with the study considered in this work, where each subject was imaged in two subsequent sessions using two different radiotracers, namely, [${}^{68}$Ga]NODAGA-RGD and [${}^{15}$O]$H_{2}O$. In [${}^{15}$O]$H_{2}O$ studies the subject is imaged feet first with respect to the scanner bore, which makes it hard to establish a line-of-sight with the RPM camera and the marker block. On the other hand, the measurement nodes of the MMS system are similar to ECG patches, and do not cause logistical difficulties during setup of the [${}^{15}$O]$H_{2}O$ imaging study. Secondly, in the [${}^{68}$Ga]NODAGA-RGD reconstructions presented in Section 3.3 we used quantile-based amplitude gating. Such gating is by design invariant to the multiplication of the respiration signal by a positive constant. Therefore, uncalibrated and calibrated MMS respiration signals will result in the same gated PET images, which makes the comparison between calibrated MMS and RPM signals fair.

### 4.3. Applicability to Respiratory-Gated PET Studies

Based on the gating statistics and image quality analysis (Table 5, Table 6, Table 7, Table 8 and Table 9), MMS respiratory-gated PET images achieved comparable gating and image quality statistics as compared to the RPM-gated PET images. Both MMS and RPM were used for gating in a similar manner, and the signals were gated using identical methods, which allowed a fair comparison. The same respiratory cycles were rejected for both signal based on the RPM. The remaining differences in both the gating statistics and image quality were thus the results of the individual variations in both MMS and RPM signals across the subjects. Of note is that we rejected also the MMS cycles based on the valid cycles from the RPM signal. Using a MMS signal-based cycle rejection algorithm could improve the SNR and CV metrics. This is due to the fact that not all of the cycles considered non-valid by the RPM are in reality non-valid also for the MMS signal. However, this method was used, as it made the comparison fairer between the RPM and the MMS.

We detected a banana artifact in the lung–heart and liver–lung regions in both the RPM and the MMS-gated PET images corresponding to subject 7. The artifact was more pronounced with the MMS respiratory signal, owing to a difference in respiratory phase between the RPM and the MMS signals. The artifact was due to mismatch in CT-PET, as non-gated CT acquisition was used for attenuation correction of PET. A respiratory-gated CT acquisition collected simultaneously to the RPM, and the MMS signal acquisition should be applied for optimum attenuation correction.

It should be noted that while gating is effective against motion artifacts in PET imaging, the reduction of data used for reconstruction results in increased level of noise in images. Such noise could be mitigated using several post-processing approaches, such as deep-learning-based denoising methods [30] and fuzzy image processing methods [31,32,33].

### 4.4. Real-Time Operation Capability

To conclude this discussion, we note that our implementation of the respiration estimation algorithm is based mainly on causal signal processing techniques in order to extend it to real-time applications. The only major exception to this is the use of forward-backward Butterworth filtering in the computation of the polarity and magnitude signal. In future, we will investigate how to replace these non-causal filters with causal ones. The computational time for the MMS respiration signal was approximately 3% of the signal acquisition time with Python 3.7.9 and Numpy 1.19.1 on an Intel Core i7-4770 CPU @ 3.40 GHz with 16 GB of RAM, which shows that the computational complexity of the presented algorithm should not pose an issue for a real-time implementations. In terms of latency, it is beneficial that the delay of the shape signal is relatively low—approximately 0.4 s with the moving average window length $w_{\mathrm{avg}}^{S}$ used in this work.

## 5. Conclusions

In this work we have presented a novel method for estimating respiratory motion using MEMS motion sensors. We used a measurement system comprising of four MEMS motion sensor nodes, and estimated the respiratory motion from signals acquired from the sternum and the abdomen. Our results show that the method performs similarly or better than most of the other motion sensor-based methods for breathing frequency estimation. Furthermore, we have showed that the method can be used to obtain respiratory signal for gated PET study; the image quality and data statistics were similar to those obtained with a clinically used solution.

## Figures and Tables

**Table 1:** Summary of the population characteristics of this study in terms of the mean and the standard deviation (SD).

	No. Subjects	Age (Y)	Weight (kg)	Height (m)	Dose (MBq)
Mean	8 (8 males)	64.3	89.5	1.75	197.5
SD		8.1	16.3	0.06	19.0

**Table 2:** Parameters used for computing the MMS respiration signal results presented in this work.

Algorithm Block	Parameter	Symbol	Unit	Value
All	Downsampled sampling frequency	$f_{s}$	[Hz]	100.0
Shape	Moving average window length	$w_{\mathrm{avg}}^{S}$	[sample]	$\left\lfloor 0.8f_{s}\right\rfloor$
Shape	Circular buffer size	$N_{\mathrm{circ}}$	[sample]	$\left\lfloor 60.0f_{s}\right\rfloor$
Pol. & Magn.	Gravitational acceleration	*g*	[$m/s^{2}$]	9.81
Pol. & Magn.	Cutoff frequency	$f_{B}$	[Hz]	0.2
Fusion	Moving average window length	$w_{\mathrm{avg}}^{F1}$	[sample]	$\left\lfloor 10.0f_{s}\right\rfloor$
Fusion	Moving average window length	$w_{\mathrm{avg}}^{F2}$	[sample]	$\left\lfloor 60.0f_{s}\right\rfloor$
Fusion	Moving average window length	$w_{\mathrm{avg}}^{F3}$	[sample]	$\left\lfloor 60.0f_{s}\right\rfloor$

**Table 3:** Mean signal magnitudes for RPM and MMS respiration signals, the polarity of the respiration signal (*q*), the mean absolute error of the MMS signal with respect to RPM signal and the mean absolute breathing error rate per minute for the MMS signal with respect to RPM for the considered 8 subjects.

Subj.	RPM Magn. (cm)	MMS Magn. (cm)	Pol.	MAE Amp. (cm)	MAE fB
1	0.52	0.67	−1.0	0.17	0.62
2	0.97	0.65	1.0	0.23	0.78
3	1.14	1.03	1.0	0.23	0.69
4	1.74	1.18	1.0	0.18	0.20
5	0.68	1.00	−1.0	0.16	0.36
6	0.68	1.10	1.0	0.20	0.49
7	1.26	1.02	−1.0	0.46	0.28
8	1.17	1.24	−1.0	0.26	0.09
Mean (±SD)				0.24 (±0.09)	0.44 (±0.23)

**Table 4:** Comparison between recent MEMS-based breathing rate estimation methods in terms of the mean absolute breathing rate error per minute.

Article	Test Subject Group	MAE fB (1/min)
This work	8 subjects with acute myocardial infarction	0.44 (±0.23)
[6]	8 healthy volunteers	0.5 (±0.6)
[10]	1 post-op. patient overnight measurement	0.38 for 45% of data
[15]	7 healthy volunteers	0.768
[16]	15 healthy volunteers	0.7 (±1.0)
[17]	8 healthy volunteers	1.00 (±1.24)
[20]	15 neuromuscular patients	0.79

**Table 5:** Gating statistics in the PET images with respiratory motion-compensated reconstruction based on the MMS respiratory signal, compared to a non-gated reconstruction (100%). Lost data signifies the amount of data rejected due to cycle rejection and signal falling out of gating threshold bounds.

Subj.	Bin 1	Bin 2	Bin 3	Bin 4	Bin 5	Lost Data
1	9.57%	13.74%	14.93%	12.45%	9.65%	39.66%
2	10.27%	11.98%	13.58%	12.58%	11.79%	39.80%
3	7.81%	10.35%	13.96%	15.62%	12.38%	39.90%
4	9.67%	9.05%	9.85%	12.32%	19.17%	39.94%
5	9.59%	9.22%	11.47%	13.10%	16.63%	40.00%
6	10.94%	11.95%	12.73%	13.02%	11.77%	39.59%
7	11.01%	11.42%	11.90%	13.14%	12.53%	40.00%
8	3.40%	4.00%	5.86%	15.01%	32.34%	39.39%
Mean	9.03%	10.21%	11.78%	13.40%	15.78%	39.78%
SD	2.33%	2.76%	2.69%	1.15%	6.87%	0.20%

**Table 6:** Gating statistics in the PET images with respiratory motion-compensated reconstruction based on the RPM respiratory signal, compared to a non-gated reconstruction (100%).

Subj.	Bin 1	Bin 2	Bin 3	Bin 4	Bin 5	Lost Data
1	6.83%	9.63%	12.68%	14.52%	16.78%	39.56%
2	9.74%	11.24%	10.99%	13.27%	14.90%	39.86%
3	10.22%	12.60%	12.16%	12.80%	12.35%	39.87%
4	9.70%	9.71%	10.27%	12.00%	18.44%	39.89%
5	8.18%	9.31%	11.07%	15.03%	16.43%	39.98%
6	7.83%	9.96%	16.67%	13.02%	12.54%	39.98%
7	20.30%	7.13%	8.54%	10.33%	13.74%	39.96%
8	13.96%	5.28%	6.06%	13.88%	29.37%	31.45%
Mean	10.84%	9.36%	11.05%	13.11%	16.82%	38.82%
SD	4.10%	2.13%	2.90%	1.39%	5.15%	2.79%

**Table 7:** Image quality metrics corresponding to non-gated reconstructions. CR = contrast ratio, SNR = signal to noise ratio, CV = coefficient of variation, CNR = contrast to noise ratio.

Subj.	CR	SNR	CV	CNR
1	0.980296	17.02421	0.238911	−0.34218
2	0.817972	8.759339	0.177112	−1.94926
3	0.839961	10.3916	0.234946	−1.97993
4	0.926529	13.84539	0.236123	−1.09789
5	0.933141	12.11581	0.204299	−0.86809
6	0.807438	14.14016	0.283288	−3.37222
7	0.871556	9.918627	0.192966	−1.46174
8	0.784991	9.227095	0.461774	−2.52731
Mean	0.870236	11.92778	0.253677	−1.69983
SD	0.065287	2.699771	0.084443	0.906661

**Table 8:** Image quality metrics corresponding to MMS-based respiratory-gated reconstructions. CR = contrast ratio, SNR = signal to noise ratio, CV = coefficient of variation, CNR = contrast to noise ratio.

Subj.	CR	SNR	CV	CNR
1	0.996192	3.685625	0.394421	−0.01409
2	0.805106	2.726002	0.358326	−0.65989
3	0.798347	2.833864	0.356364	−0.7158
4	0.953703	5.678192	0.32288	−0.27564
5	0.945216	4.246757	0.301181	−0.24614
6	0.86365	5.090021	0.327836	−0.80359
7	0.810481	3.731079	0.399166	−0.87246
8	0.797792	6.173485	0.59685	−1.56472
Mean	0.871311	4.270628	0.382128	−0.64404
SD	0.076417	1.191511	0.087169	0.450218

**Table 9:** Image quality metrics corresponding to RPM-based respiratory-gated reconstructions. CR = contrast ratio, SNR = signal to noise ratio, CV = coefficient of variation, CNR = contrast to noise ratio.

Subj.	CR	SNR	CV	CNR
1	0.938885	4.076106	0.338815	−0.26532
2	0.789202	2.50273	0.33396	−0.66849
3	0.828536	2.714587	0.360038	−0.56178
4	0.989893	8.167109	0.288466	−0.08339
5	0.936245	4.774381	0.309522	−0.32512
6	0.891802	4.701099	0.342195	−0.57036
7	0.893655	4.872468	0.280067	−0.57982
8	0.806291	6.709808	0.569189	−1.61201
Mean	0.884314	4.814786	0.352782	−0.58329
SD	0.066342	1.773032	0.085762	0.43067

**Table 10:** Mean absolute error in amplitude (cm). First row: non-calibrated MMS vs. RPM. Second row: calibrated MMS vs. baseline drift corrected RPM.

Subj.	1	2	3	4	5	6	7	8	Mean (±SD)
Non-calib.	0.17	0.23	0.23	0.18	0.16	0.20	0.46	0.26	0.24 (±0.09)
Calib.	0.08	0.13	0.14	0.08	0.05	0.11	0.10	0.08	0.10 (±0.0008)

## Abbreviations

The following abbreviations are used in this manuscript:

MEMS	Microelectromechanical Systems
IMU	Inertial Measurement Unit
PET	Positron Emission Tomography
CT	Computed Tomography
ECG	Electrocardiography
MMS	Motion Measurement System
RPM	Real-time Position Management
PCA	Principal Component Analysis
ICA	Independent Component Analysis
VOI	Volume of Interest
CR	Contrast Ratio
SNR	Signal to Noise Ratio
CV	Coefficient of Variation
CNR	Contrast to Noise Ratio
